# APTES-Modified SBA-15 as a Non-Toxic Carrier for Phenylbutazone

**DOI:** 10.3390/ma15030946

**Published:** 2022-01-26

**Authors:** Adrianna Dadej, Aneta Woźniak-Braszak, Paweł Bilski, Hanna Piotrowska-Kempisty, Małgorzata Józkowiak, Maciej Stawny, Daniela Dadej, Michał Mrotek, Anna Jelińska

**Affiliations:** 1 Department of Pharmaceutical Chemistry, Faculty of Pharmacy, Poznan University of Medical Sciences, Grunwaldzka 6, 60-780 Poznań, Poland; mstawny@ump.edu.pl (M.S.); 74860@student.ump.edu.pl (M.M.); ajelinsk@ump.edu.pl (A.J.); 2 Functional Materials Physics Division, Faculty of Physics, Adam Mickiewicz University, Uniwersytetu Poznańskiego 2, 61-614 Poznań, Poland; abraszak@amu.edu.pl; 3 Medical Physics and Radiospectroscopy Division, Faculty of Physics, Adam Mickiewicz University, Uniwersytetu Poznańskiego 2, 61-614 Poznań, Poland; bilski@amu.edu.pl; 4 Frank Laboratory of Neutron Physics, Joint Institute for Nuclear Research, 141980 Dubna, Russia; 5 Department of Toxicology, Faculty of Pharmacy, Poznan University of Medical Sciences, Dojazd 30, 60-631 Poznań, Poland; hpiotrow@ump.edu.pl (H.P.-K.); malgorzata.jozkowiak@gmail.com (M.J.); 6 Department of Endocrinology, Metabolism and Internal Diseases, Faculty of Medicine, Poznan University of Medical Sciences, Przybyszewskiego 49, 60-355 Poznań, Poland; daniela.dadej@student.ump.edu.pl

**Keywords:** dissolution rate, SBA-15 mesoporous silica, drug delivery system, phenylbutazone, physicochemical techniques

## Abstract

Improvement of the bioavailability of poorly soluble medicinal substances is currently one of the major challenges for pharmaceutical industry. Enhancing the dissolution rate of those drugs using novel methods allows to increase their bioavailability. In recent years, silica-based mesoporous materials have been proposed as drug delivery systems that augment the dissolution rate. The aim of this study was to analyse the influence of phenylbutazone adsorption on SBA-15 on its dissolution rate. Moreover, we examined the cytotoxicity of the analyzed silica. The material was characterized by SEM, TEM, DSC, ^1^H-NMR, XRD, and FT-IR. The phenylbutazone did not adsorb on unmodified SBA-15, while the adsorption on APTES-modified SBA-15 resulted in 50.43 mg/g of loaded phenylbutazone. Phenylbutazone adsorbed on the APTES-modified SBA-15 was then released in the hydrochloric acidic medium (pH 1.2) and phosphate buffer (pH 7.4) and compared to the dissolution rate of the crystalline phenylbutazone. The release profiles of the amorphous form of adsorbed phenylbutazone are constant in different pH, while the dissolution rate of the crystalline phenylbutazone depends on the pH. The cytotoxicity assays were performed using the Caco-2 cell line. Our results indicate that the analyzed material ensured phenylbutazone adsorption in an amorphous state inside the mesopores and increased its dissolution rate in various pH levels. Furthermore, the cytotoxicity assay proved safety of studied material. Our study demonstrated that APTES-modified SBA-15 can serve as a non-toxic drug carrier that improves the bioavailability of phenylbutazone.

## 1. Introduction

Silica mesoporous materials were first synthetized in 1992 by scientists from Mobil Oil [1]. In 1998, Zhao et al. obtained a new type of mesoporous material—SBA (Santa Barbara Amorphous). SBA-15 material presents a highly ordered and a two-dimensional, hexagonal structure. Compared to the previously obtained MCM-41 (Mobil Composition of Matter No. 41) material, the walls of SBA-15 are thicker, so that the material exhibits greater thermal and mechanical stability [2]. Moreover, the specific surface area of SBA-15 is increased due to the presence of additional micropores in the walls of the material [3]. Over the years, further modifications of the material synthesis have been developed in order to obtain the desired properties of silicas.

The silanol groups on the surface of mesoporous materials allow surface functionalization. The incorporation of organic residues enables to obtain the required material properties. Surface modifications facilitate, among others, the acquisition of acidic, basic, or optical properties, increasing the adsorption efficiency of molecules and modifying their release profiles in the organism (time and site of release) [4].

Mesoporous silicas can serve as drug carriers. The first successful adsorption of ibuprofen on MCM-41 was presented by Vallet-Regi [5]. Further studies demonstrated that SBA-15 material increases the dissolution rate of sparingly soluble medicinal substances. Adsorption of molecules on SBA-15 silica allows the stabilization of their amorphous state, preventing the transition into a crystalline state. Compounds in the amorphous form are characterized by a reduced lattice energy, resulting in a greater dissolution rate and higher bioavailability. Furthermore, the hydrophilic nature of the mesoporous silica surface facilitates the wetting and dispersion of the adsorbed molecules, accelerating their dissolution [6]. Dissolution rate improvement was confirmed for various active substances, such as ampicillin, amoxicillin, emodin, itraconazole, or naproxen [7,8,9,10,11]. In addition, mesoporous materials based on SBA-15 are also used as catalysts in organic synthesis following the principles of green chemistry [12].

Phenylbutazone is a non-steroidal anti-inflammatory drug, classified as BCS II (Biopharmaceutics Classification System), characterized by good permeability but low solubility. It was approved by FDA in the middle of the last century, mainly for the treatment of rheumatoid arthritis. However, side effects, such as gastrointestinal and haematological disorders, including aplastic anemia, considerably limited the use of this drug in humans. Still, however, phenylbutazone is widely used in veterinary medicine as an analgesic and anti-inflammatory agent in horses [13]. 

There are several reports in the literature concerning methods used to enhance the dissolution rate of phenylbutazone. Until now, it was proposed to prepare solid dispersions with polyethylene glycol 8000 [14], formulate Syloids silica-based formulations [15] and adsorb the drug on non-modified SBA-15 silica [16]. 

The aim of this study was to analyze the adsorption process of phenylbutazone on the APTES-modified mesoporous silica SBA-15 and to evaluate the modification of the phenylbutazone release profile from the carriers in comparison with the crystalline substance. Additionally, we characterized obtained materials by nitrogen adsorption/desorption analysis, SEM and TEM imaging, and methods, such as XRD and DSC. We also investigated the effect of drug adsorption on modified silica on its cytotoxicity.

## 2. Materials and Methods

### 2.1. Chemicals and Materials

Tetraethyl orthosilicate (TEOS) (≥99.0%), sodium dodecyl sulfate (≥99.0%), hydrochloric acid (purum p.a. ≥32.0%), Pluronic^®^P-123, (3-aminopropyl)triethoxysilane (APTES) (99%), and anhydrous toluene (99.8%) were supplied from Sigma-Aldrich Co., (St Louis, MO, USA). Chloroform (p.a. ≥98.5%), propan-2-ol (p.a. ≥99.7%), dipotassium phosphate (pure p.a.), sodium chloride (pure p.a.), analytical weight of hydrochloric acid 0.1 mol/L (0.1 N), and sodium hydroxide 0.1 mol/L (0.1 N) were purchased from Avantor Performance Materials Poland (Gliwice, Poland). Phenylbutazone (>98%) was supplied from Tokyo Chemical Industry CO (Zwijndrecht, Belgium).

#### Mesoporous Material Preparation and Surface Modification

The SBA-15 mesoporous silica was synthesized in accordance with the protocol described by Zhao et al. [2], with modifications presented in our previous study [17]. The SBA-15 surface modification process was carried out using the grafting method [17]. Briefly, we dried the silica for 24 h at 110 °C. Then, we mixed 3.0 g of silica with 50 mL of APTES ((3-aminopropyl) triethoxysilane) solution in toluene (0.10 mol/L). Next, we heated the mixture at 100 °C for 24 h. Then, we filtered the obtained product and rinsed it with anhydrous toluene (5 × 75 mL) and with chloroform (5 × 75 mL). We dried the modified material at 40 °C for 3 h, and at 80 °C for next 24 h, to remove the remaining organic solvent. The APTES-modified SBA-15 was designated as SBA-15-pr-NH_2_.

### 2.2. Phenylbutazone Adsorption Studies

We conducted the adsorption process of phenylbutazone (PBZ) on SBA-15 and SBA-15-pr-NH_2_ as follows. We prepared the 3.0 mg/mL PBZ solution in 2-propanol and combined 5.0 mL of prepared solution with 50.0 mg of SBA-15 or SBA-15-pr-NH_2_ silica. Then, the mixture was stirred for 48 h at 25 °C to achieve adsorption equilibrium state. Next, we centrifuged the suspension at 6000 rpm for 15 min. The obtained precipitate was dried at 50 °C for 48 h. We did not obtain adsorption on unmodified material. Samples with adsorbed PBZ on APTES-modified SBA-15 were designated as SBA-15-pr-NH_2_:PBZ. 

We calculated the amount of adsorbed PBZ as the difference in concentration of the solutions before and after the adsorption. We evaluated the solutions’ concentrations using the UV/VIS LAMBDA 20 Perkin Elmer (PerkinElmer, Inc., Waltham, MA, USA) spectrophotometer at 240 nm.

In order to calculate the drug loading efficiency, we used the following formula:

(1) $$\mathrm{drug}\;\mathrm{loading}\;\mathrm{efficiency}\;(\%)=\frac{\mathrm{weight}\;\mathrm{of}\;\mathrm{PBZ}\;\mathrm{in}\;\mathrm{the}\;\mathrm{sample}\;\left(\mathrm{mg}\right)}{\mathrm{weight}\;\mathrm{of}\;\mathrm{the}\;\mathrm{carrier}\;\mathrm{in}\;\mathrm{the}\;\mathrm{sample}\;\left(\mathrm{mg}\right)}\;\times \;100\%.$$

### 2.3. Characterization Methods

#### 2.3.1. Powder X-ray Diffraction (XRD)

The small-angle X-ray powder diffraction patterns were performed with Bruker D8 Advance (Billerica, MA, USA) over the range 0.6° < 2θ < 8.0° with a scan speed 0.02°/1–2 s. Wide-angle X-ray powder diffraction patterns were recorded with Bruker D2 Phaser (Billerica, MA, USA) over the range 5.0° < 2θ < 45.0° with a scan speed 0.02°/1–2 s.

#### 2.3.2. Differential Scanning Calorimetry (DSC)

Differential scanning calorimetry (DSC) thermograms were recorded using DSC 214 Polyma Netzsch (Netzsch Group, Selb, Germany). All samples weighing approximately 5 mg were scanned from 25 to 170 °C at a scanning rate of 5 °C/min under nitrogen gas flow (30 mL/min).

#### 2.3.3. Nitrogen Adsorption/Desorption

Nitrogen adsorption/desorption isotherms were determined at −196 °C by Autosorb iQ analyzer (Quantachrome Instruments, Boynton Beach, FL, USA). The samples of pure silica and materials with adsorbed PBZ were degassed in vacuum for 24 h at 100 °C and room temperature, respectively. The specific surface area was obtained by BET (Brunauer-Emmett-Teller) isotherm. The size distribution, pore volume and diameter were estimated from the desorption branch of the isotherms in accordance with BJH (Barret-Joyner-Halenda) method.

#### 2.3.4. Transmission and Scanning Electron Microscopy (TEM/SEM)

TEM images were recorded by a JOEL JEM 1200 EX electron microscope (JEOL Ltd., Tokyo, Japan). operated at 80 kV. SEM images were taken on a Zeiss EVO-40 electron microscope (Carl Zeiss AG, Oberkochen, Germany).

#### 2.3.5. Fourier Transformed Infrared Spectroscopy (FTIR)

The FTIR spectra were collected using a Bruker FTIR IFS 66/s spectrometer (Billerica, MA, USA) in the range 400–4000 cm^−1^ (the resolution of 1 cm^−1^) with the KBr pellet technique (1 mg of the studied material and 200 mg of KBr).

#### 2.3.6. Spectrophotometry

The UV/VIS spectra were performed on UV/VIS LAMBDA 20 Perkin Elmer spectrophotometer. The UV method was validated. The selectivity, linearity, precision, and accuracy were set up according to the ICH Q2(R1) [18]. In order to fit the best statistical model of the relationships between absorbance and concentration of PBZ, the homoscedasticity of data were checked using F-test. According to the literature [19,20,21], it is suggested to evaluate the fit of the ordinary least squares linear (OLS) regression by assessing the homoscedasticity of data and, in the case of heteroscedasticity, search for other models, which better defined the correlation between the concentration and the response of the detector [21,22]. When the homogeneity was not found, the homoscedasticity was evaluated by plotting graphs of the residuals versus concentration. The residuals were established by the difference between the obtained data at each point of the calibration curve and the calculated values from the OLS equation. Given the evidence of heteroscedasticity, the weighted WLS was utilized to calculate the regression line that unifies the differences between errors throughout the working range. In the course of calculations, the appropriate weighting factors, w_i_, i.e., $\frac{1}{y^{0.5}}$, $\frac{1}{y}$, $\frac{1}{y^{2}}$, $\frac{1}{x^{0.5}}$, $\frac{1}{x}$, and $\frac{1}{x^{2}}$, were used. Subsequently, the sums of the percentage relative errors (∑ %RE) were calculated for each model to determine the best fitting weighted regression line.

#### 2.3.7. Solid-State ^1^H-NMR

Solid-state ^1^H-NMR measurements were carried out on a pulse spectrometer operating at a frequency of 25 MHz (El-Lab Tel-Atomic, Jackson, MI, USA) [17]. The samples of PBZ and SBA-15-pr-NH_2_:PBZ were sealed in the glass tubes and degassed. Measurements were performed in a temperature range from 80 K to 300 K. The proton spin-lattice relaxation times T_1_ in the laboratory frame were estimated by fitting the equation: M_z_(t) = M_0_*(1 − exp(−t/T_1_), where: M_0_ is the equilibrium magnetization, and T_1_ is the relaxation time, to the experimental data obtained using a conventional saturation method. The measurement error was around 3%. For PBZ and SBA-15-pr-NH_2_:PBZ, the recovery of magnetization M_z_(t) was one-exponential in the entire temperature range.

### 2.4. Drug Release Studies

We carried out the in vitro PBZ release studies from SBA-15-pr-NH_2_:PBZ and the dissolution tests of pure PBZ on an Electrolab EDT 08Lx apparatus (Electrolab, Janki Impex, Gujarat, India) using a rotating paddle method. During the test, the temperature was maintained at 37 °C, and the mixing speed was set at 70 rpm. In each analysis, we used silica samples corresponding to 4 mg of PBZ or the same amount of pure compound. We performed each test in triplicate. We limited the amount of the fluid at 500 mL of hydrochloric acidic medium at pH 1.2 ± 0.1 and phosphate buffer at pH 7.4 ± 0.1 (Ph. Eur. X) in order to maintain the sink conditions. After 5, 15, 30, 60, 90, 120 min, and after 3, 4, 5, 6, and 24 h, we removed 4 mL of the liquid from the vessel and filtered it through a 0.22 µm PTFE filters. We replenished the diminished fluid in the vessel with fresh hydrochloric acidic medium at pH 1.2 ± 0.1 or the phosphate buffer at pH 7.4 ± 0.1 in the appropriate volume. We measured the PBZ concentration by UV spectrophotometry at 236 nm. Each measurement of the samples in the phosphate buffer was preceded by the addition of 0.3 mL of 1 mol/L HCl in order to avoid the occurrence of keto-enol tautomerism.

In order to compare the obtained release profiles, we used a method based on the calculation of the similarity coefficients *f*_2_. We calculated the similarity factors *f*_2_ according to Equation (2) [23]:

(2) $$f_{2}=50*\;\log_{\;}\{[1+\frac{1}{n}\;\sum_{t=1}^{n}\left(R_{t}-T_{t}\right){\;}^{2}]{\;}^{-0.5}*100\},$$

where *n* is number of sample points, and *R_t_* and *T_t_* are the percentage of dissolved PBZ from the reference or released PBZ from the samples, respectively, at time *t.*

### 2.5. Cytotoxicity Study

#### 2.5.1. Cell Culture 

Caco-2 human colon adenocarcinoma cell line was obtained from the European Type Culture Collection (Sigma-Aldrich Co., St Louis, MO, USA). The cell line was maintained in phenol-free DMEM medium (Sigma-Aldrich Co., St Louis, MO, USA), supplemented with 10% foetal bovine serum (FBS), streptomycin (0.1 mg/mL), penicillin (100 U/mL), 1% non-essential amino acids mixture, and 2 mM glutamine (Sigma-Aldrich Co., St Louis, MO, USA). The assays were carried out with Caco-2 cells from the 20th passage. 

#### 2.5.2. Cell Viability 

Caco-2 cells were cultivated under the standard conditions at 37 °C in a humidified atmosphere containing 95% air and 5% CO_2_. To evaluate the effects of PBZ, SBA-15-pr-NH_2_ and SBA-15-pr-NH_2_:PBZ on cell viability, confluent stock cultures were detached using trypsin and seeded in 96-well plates at a density of 2 × 10^4^ cells/well in 150 µL of growth medium. The cells were allowed to attach, and, after 48 h, we added the analyzed compounds. In the assays, we investigated the concentrations of SBA-15-pr-NH_2_ in the range of 0.125–1.0 mg/mL and the PBZ concentrations corresponding to the amount of the PBZ adsorbed on the SBA-15-pr-NH_2_ used for the assays. After 2 h of incubation, we measured the cell viability using the CellTiter-Glo^®^ Luminescent Cell Viability Assay (Promega, Madison, WI, USA), according to the manufacturer’s instruction.

### 2.6. Statistical Analysis

The data were analyzed using Statistica 12 software (StatSoft, Cracow, Poland). One-way analysis of variance (ANOVA) was used to determine the statistical significance occurring among the samples. The a priori level of significance was *p* < 0.05. All measurements were conducted in triplicate.

## 3. Results and Discussion

### 3.1. Powder X-ray Diffraction

**Figure 1:**
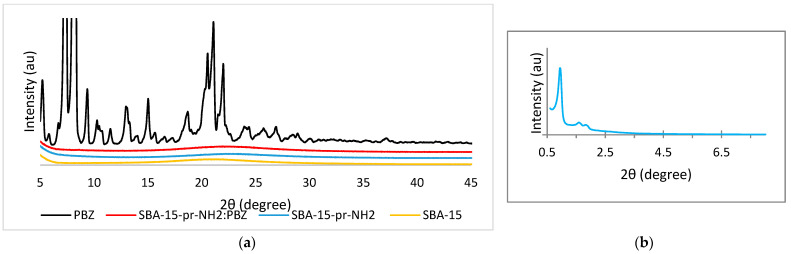
(**a**) Wide-angle XRD patterns of PBZ, SBA-15, SBA-15-pr-NH_2_:PBZ, and SBA-15-pr-NH_2_. (**b**) Small-angle XRD pattern of SBA-15-pr-NH_2_.

We used the XRD analysis to determine amorphous or crystalline state of the samples. Figure 1a presents the wide-angle diffractograms of PBZ, SBA-15, SBA-15-pr-NH_2_ and SBA-15-pr-NH_2_:PBZ. The diffraction pattern obtained for phenylbutazone shows many characteristic peaks of the PBZ in crystalline form. The typical reflection peaks for PBZ are located at diffraction angles (2θ) of 6.3°, 8.5° for main peaks and at 13.5°, 15.1°, and 21.9° [24]. In contrast, the diffraction pattern for SBA-15 and SBA-15-pr-NH_2_ shows one broad peak that confirms the presence of amorphous silica [25]. The diffractogram obtained for SBA-15-pr-NH_2_:PBZ is comparable to that of modified silica and shows no characteristic peaks of crystalline PBZ. These results confirm that adsorbed PBZ is in an amorphous state.

Figure 1b shows the small-angle diffractogram of SBA-15-pr-NH2. It reveals three well-resolved peaks, the most intense at 2 Θ ≈ 1°, corresponding to (100), (110), and (200) planes. Presented diffractogram indicates an ordered structure of modified material and hexagonal symmetry with the P6 mm space group. Our results are in line with the literature data [2,7].

### 3.2. Differential Scanning Calorimetry 

**Figure 2:**
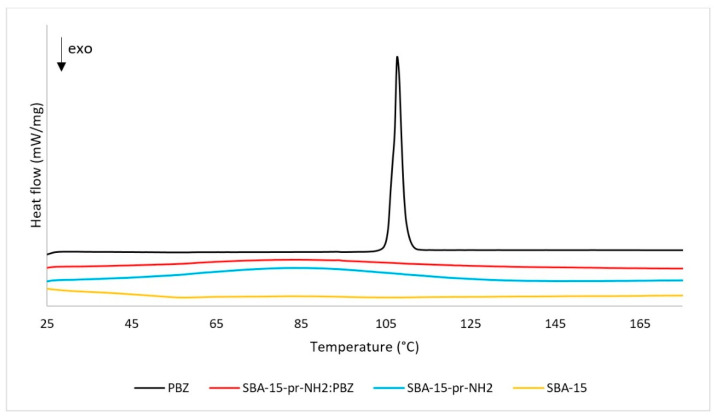
DSC thermograms of PBZ, SBA-15, SBA-15-pr-NH_2_:PBZ, and SBA-15-pr-NH_2_.

The DSC analysis supports our findings from XRD measurements. The results of the DSC assays are presented in the Figure 2. The DSC thermogram of PBZ shows one endothermic peak at 107.6 °C associated with the solid-liquid transition and confirming the crystalline state of the drug [24]. For the SBA-15 and SBA-15-pr-NH_2_, we observe a peak corresponding to glass transition at 75 °C and 65 °C, respectively, proving the amorphous state of the silicas [26]. The absence of melting or degradation peaks indicates the thermal stability of SBA-15-pr-NH_2_. Similarly, the DSC curve for SBA-15-pr-NH_2_:PBZ demonstrates no melting or decomposition peaks, which proves the amorphous state of PBZ incorporated inside the mesopores of the SBA-15-pr-NH_2_ [27].

### 3.3. Nitrogen Adsorption/Desorption

**Figure 3:**
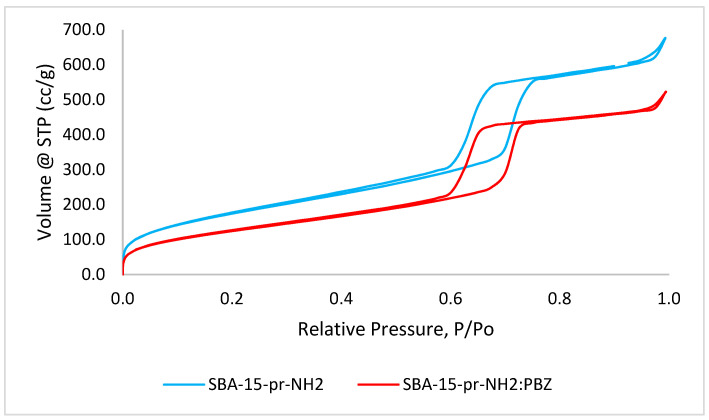
Nitrogen adsorption-desorption isotherms of SBA-15-pr-NH_2_ and SBA-15-pr-NH_2_:PBZ.

Figure 3 presents the nitrogen adsorption–desorption isotherms for SBA-15-pr-NH_2_ and SBA-15-pr-NH_2_:PBZ, and Table 1 shows their textural parameters. The mesoporous structure with microporous content of analyzed materials is confirmed by the presence of irreversible type IV isotherms with H1 hysteresis loop (IUPAC classification) [25,27]. 

Our results show that unmodified SBA-15 silica has the highest specific surface area, pore volume and pore diameter. As confirmed by earlier studies, the introduction of functional groups on the surface of silica reduces the above-mentioned parameters. Similarly, in our research, APTES-functionalization resulted in a reduction of specific surface area, volume, and diameter of the pores. It is suggested that, during surface functionalization, aminopropyl groups preferentially bind to the outer parts of mesopores and micropores, resulting in partial blockage of the pores and reduction of the aforementioned parameters [7,8]. We observed a further decrease in all values after the adsorption of PBZ to SBA-15-pr-NH_2_ [25,27].

### 3.4. Transmission Electron Microscopy

**Figure 4:**
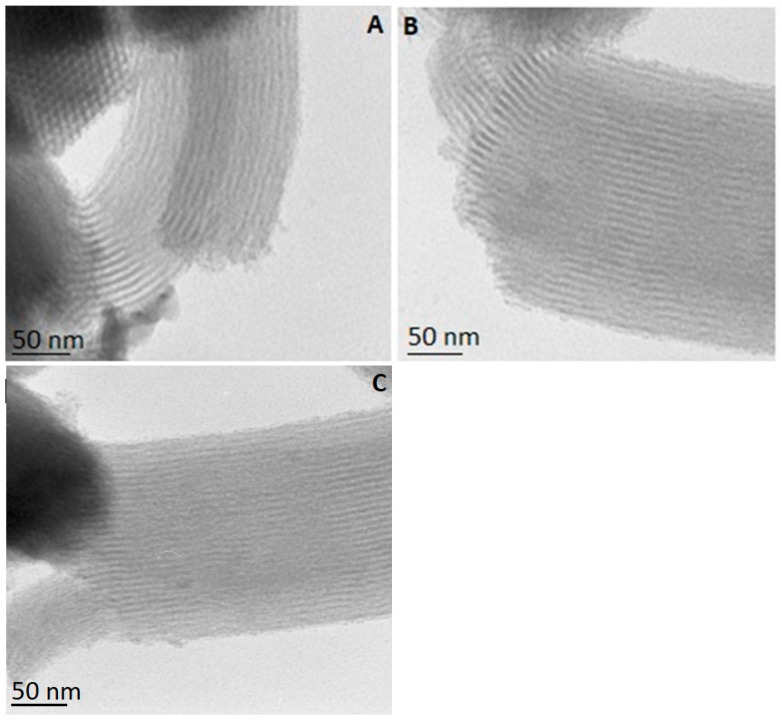
TEM images of (**A**) SBA-15-pr-NH_2_, (**B**) SBA-15-pr-NH_2_:PBZ, and (**C**) SBA-15.

We determined the structure of the SBA-15, SBA-15-pr-NH_2_, and SBA-15-pr-NH_2_:PBZ by TEM imaging (Figure 4). All micrographs present parallel mesoporous channels, which confirms the two-dimensional, ordered structure of the analyzed silicas. Noteworthy is that neither the modification of the SBA-15 with 3-aminopropyl groups nor the PBZ loading changed the structure of SBA-15-pr-NH_2_. Our results are in line with previously reported data [9,27].

### 3.5. Scanning Electron Microscopy

**Figure 5:**
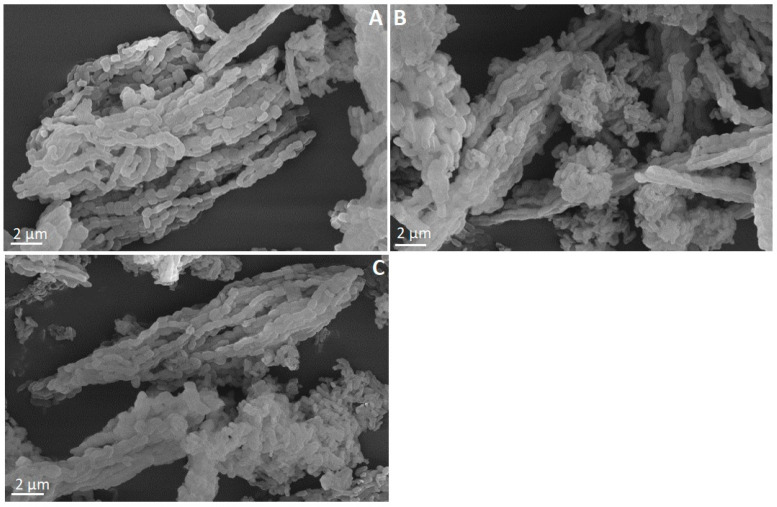
SEM images of (**A**) SBA-15-pr-NH_2_, (**B**) SBA-15-pr-NH_2_:PBZ, and (**C**) SBA-15.

We investigated the morphology of the SBA-15, SBA-15-pr-NH_2_, and SBA-15-pr-NH_2_:PBZ by SEM imaging. The SEM micrographs presented in Figure 5 show oval, well-formed rod-like particles. Individual grains with a size of about 1 µm aggregated into larger linear structures. The functionalization of the silica’s surface did not influence the morphology of analyzed silica. Micrograph B shows no additional phenylbutazone crystals, indicating drug incorporation inside the mesopores. Presented results corroborate previous findings [27,28].

### 3.6. Fourier Transformed Infrared Spectroscopy

**Figure 6:**
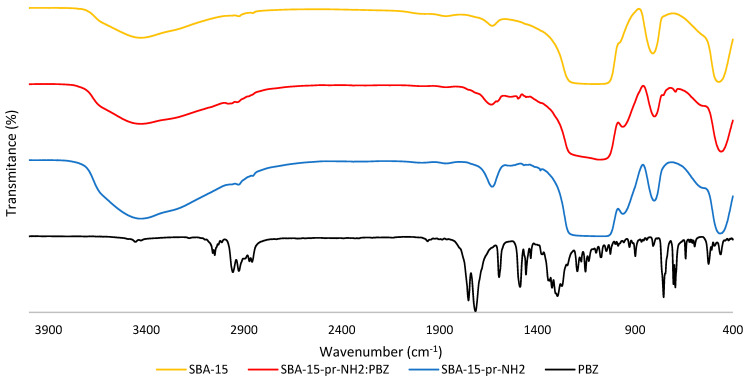
FT-IR spectra of SBA-15, SBA-15-pr-NH_2_, SBA-15-pr-NH_2_:PBZ, and PBZ.

The FT-IR spectra of PBZ, SBA-15-pr-NH_2_, and SBA-15-pr-NH_2_:PBZ are presented in Figure 6. In the spectrum of SBA-15, the broad band around 3430 cm^−1^ can be assigned to stretching vibrations of O-H groups (Si-OH), and the band at 1638 cm^−1^ to bending vibrations of O-H bonds (OH groups). In the range 1100–1200 cm^−1^, we detected the asymmetric stretching vibrations of Si-O-Si [29]. The band at 960 cm^−1^ can be attributed to free silanol groups on the surface of silica [30]. Symmetrical stretching vibrations and deformation vibrations of Si-O-Si bonds are detected at 800 cm^−1^ and at 465 cm^−1^ [31]. In the spectrum of the SBA-15-pr-NH_2_, the broad band at 3430 cm^−1^ is assigned to the stretching vibrations of the silanol groups [32]. The band at 2920 cm^−1^ can be identified as stretching vibrations from the propyl chain [11]. The peaks at 1550 cm^−1^ and 1630 cm^−1^ are identified as the N-H bending vibrations. The broad peak in the range of 1080–1200 cm^−1^ can be attributed to the Si–O–Si asymmetric stretching vibrations. This peak overlaps the C-N stretching vibrations, which are normally located in the range of 1000–1200 cm^−1^ [33]. The bands at 800 cm^−1^ and 460 cm^−1^ are assigned to the symmetric stretching and bending vibrations of Si–O–Si, respectively [32]. The above allows us to conclude that the functionalization process was carried out correctly.

In the spectrum of PBZ, bands at 1715 cm^−1^ and 1753 cm^−1^ are assigned to C=O stretching vibrations. The peak at 1595 cm^−1^ corresponds to C=C stretching vibrations of the phenyl ring. The band at 1486 cm^−1^ can be identified as C-H vibrations. The C-N bonds can be detected at 1270 cm^−1^. The bands at 754 cm^−1^ and 694 cm^−1^ are attributed to C-H vibrations [15,34,35].

The SBA-15-pr-NH_2_:PBZ spectrum displays additional peaks in comparison to SBA-15-pr-NH_2_ located at 690 cm^−1^, 1493 cm^−1^, and 1755 cm^−1^ and an increase in the intensity of the peak at 2977 cm^−1^. Additionally, the presence of the hydrogen bonds between phenylbutazone molecules and silica surface results in a broader band between 3441–3600 cm^−1^. All of the above proves the successful loading of PBZ.

### 3.7. Proton Nuclear Magnetic Resonance

**Figure 7:**
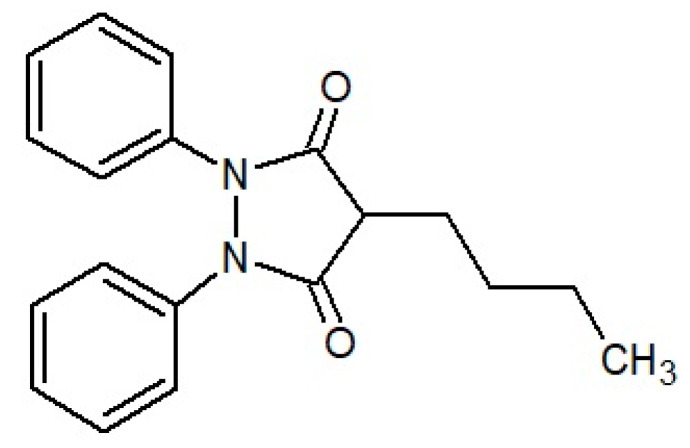
Scheme of phenylbutazone.

**Figure 8:**
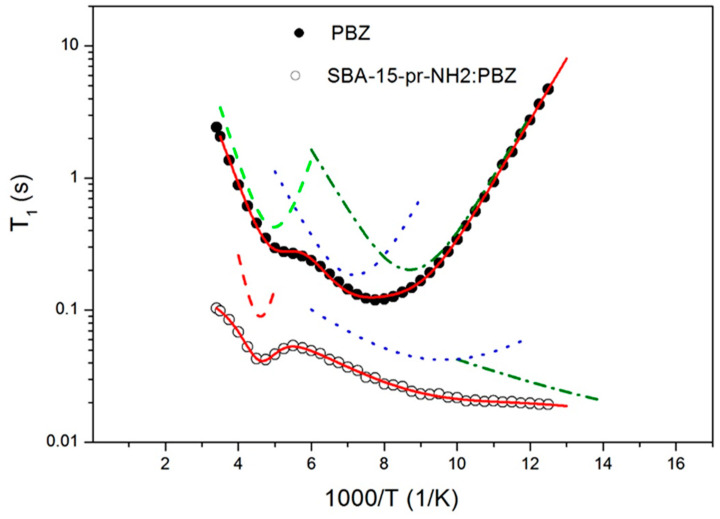
Temperature dependence of the spin-relaxation time T_1_ in the laboratory frame for PBZ and SBA-15-pr-NH_2_:PBZ.

^1^H-NMR technique allows the study of the internal dynamic of molecular groups existing in a molecule of phenylbutazone (Figure 7). Using the ^1^H-NMR technique, it is possible to confirm changes in the structure of phenylbutazone-loaded APTES-modified SBA-15 mesoporous material, as well as changes in the molecular dynamics of its molecular groups. Based on the temperature dependence, it is possible to determine the activation parameters of these molecular movements for which the theoretical description is consistent with the experimental data. Figure 8 presents the spin-lattice relaxation times T_1_ in the laboratory frame as a function of the reciprocal temperature for PBZ and SBA-15-pr-NH_2_:PBZ, respectively.

For PBZ two local minima were observed, as shown in Figure 8. The first one, in the high-temperature range, with T_1_ equal to 197 ms, appeared in the vicinity of 197 K, and the second one, in the low-temperature range, asymmetrical of T_1_ = 121 ms, was observed at 127 K. To characterize molecular motions of PBZ, the temperature dependence of the relaxation times T_1_ was analyzed in terms of dipole-dipole Bloembergen–Purcell–Pound (BPP) theory [36]. It was assumed that the T_1_ values were determined by dipolar interactions modulated by three different molecular processes: the hindered rotation of the methyl CH_3_ group around its threefold axis C3, reorientation of the butyl group, and reorientation of the whole C_4_H_9_ chain. The relaxation rate of the multi-proton system can be generally described as:

(3) $$\frac{1}{T_{1}}={\left(\frac{1}{T_{1}}\right)}^{methyl}+{\left(\frac{1}{T_{1}}\right)}^{butyl}+{\left(\frac{1}{T_{1}}\right)}^{chain},$$

where each contribution is expressed by Equation (4). The spin-lattice relaxation rate ${\left(\frac{1}{T_{1}}\right)}^{*}$ is given by the following formula [37,38]:

(4) $${\left(\frac{1}{T_{1}}\right)}^{*}=\frac{2}{3}\gamma ∆M_{2}\left(\frac{\tau_{c}}{1+\omega_{0}^{2}\tau_{c}^{2}}+\frac{4\tau_{c}}{1+4\omega_{0}^{2}\tau_{c}^{2}}\right),$$

where: ∗ concerns the relaxation of the ethyl group, the butyl group, and the whole chain *γ* is a gyromagnetic ratio of protons, and Δ*M*_2_ is a reduction of the second moment, the correlation time is given by Arrhenius formula:

(5) $$\tau_{c}=\tau_{0}\exp\frac{E_{a}}{RT}.$$

A wide and asymmetric minimum of 121 ms at about 127 K of *T*_1_ = 121 ms, depicted in Figure 8, was attributed to the hindered rotation of the methyl CH_3_ group around its threefold axis C3. In the higher temperature region, the occurrence of reorientation of the butyl group was assumed, and the sharp minimum was attributed to the reorientation of the whole chain. It was approximated that the proton spin-lattice relaxation time due to these motions is given by the same Formula (4).

The best numerical fit of Equations (3) and (4) to the experimental relaxation times in the whole temperature range is presented as solid lines in Figure 8, while the partial contributions are presented as dotted lines. The activations parameters are summarized in Table 2.

For the PBZ, the methyl group reorientation is characterized by the activation energy *E_a_* of 8.8 kJ/mol and a *τ*_0_ value of 3.9 × $10^{-13}$ s, which is consistent with the literature data [39]. 

The reorientation of the butyl group is described by activation energy *E_a_* = 9.8 kJ/mol and the correlation time *τ*_0_ = 8.2 × 10^−13^ s. For the motion of the whole chain C_4_H_9_, the following activation parameters are estimated: activation energy *E_a_* =16 kJ/mol, correlation time *τ*_o_ = 3.8 × 10^−13^ s.

For the SBA-15-pr-NH_2_:PBZ, the analysis of ^1^H-NMR data was performed according to the procedure described for pure PBZ, and the activation parameters of the assumed molecular processes were extracted and are shown in Table 2. For PBZ incorporated into APTES-modified mesoporous silica, different temperature dependence of the relaxation times, T_1_, was obtained in comparison to a pure phenylbutazone drug. 

Shorter relaxation times mean faster relaxation of phenylbutazone incorporated into APTES-modified SBA-15 mesoporous material, which is characteristic of amorphous systems. In the temperature dependence of the relaxation times, only a narrow minimum of 39 ms is observed at 216 K. The shift of this minimum, assigned to the reorientation of the whole chain of PBZ, towards higher temperatures proves that molecular dynamics of this group of PBZ is hindered inside the pores. In addition, the estimated activation energy of the motion of this group is higher compared to a pure drug and is equal to 24.8 (kJ/mol). For the SBA-15-pr-NH_2_:PBZ, no other minimum was observed at the low temperatures.

However, by fitting Equations (3) and (4) to the experimental data, the activation parameters of two additional molecular movements were determined. It was suggested that motion, which is characterized by the relaxation times *τ*_02_ = 5.8 × $10^{-11}$ (s) and the activation energy *E_a_*_2_ = 3.7 (kJ/mol) can be associated with local motion of the methyl group. The third low temperature motion described by the relaxation time *τ*_03_= 7.8 × $10^{-11}$ (s) and low activation energy *E_a_*_3_= 1.8 (kJ/mol) may be related to the jumps of the hydrogen atom in hydrogen bonds, which arise as a result of the interaction of the NH_2_ amine groups of the modified silica with the incorporated drug [17].

The ^1^H-NMR results confirm that loaded phenylbutazone in the SBA-15 mesopores undergoes structural rearrangements upon transition from the crystalline state to the amorphous one, which improves the bioavailability of the drug [40,41].

### 3.8. Validation of UV Method

The method was linear in the range of 0.003–0.03 mg/mL, and the relationships between absorbance and concentration calculated based on OLS and WLS were characterized by a high correlation coefficient (Table 3). The homoscedasticity test of the data was performed in order to evaluate the fit of the OLS model, and it was found that the F_calc_ was greater than the F_crit_, and the residues gave a wide distributed band of values around the axis of concentration. That evidence implied the heteroscedasticity of the data and the need for using the WLS to calculate the weighted regression parameters. Six empirical weights, $\frac{1}{y^{0.5}}$, $\frac{1}{y}$, $\frac{1}{y^{2}}$, $\frac{1}{x^{0.5}}$, $\frac{1}{x}$, and $\frac{1}{x^{2}}$, were used to convert the regression equation to the weighted regression equation. The obtained values of equation coefficients slope (a) and intercept (b), and correlation coefficient (r), are presented in Table 3. The best-fitting model was determined by comparing the sums of percentage relative errors (∑%RE) and were as follows: $\frac{1}{y}$ for PBZ in isopropanol, and $\frac{1}{y^{0.5}}$ for PBZ in HCl medium and phosphate buffer.

The accuracy and limit of detection (LOD) and quantification (LOQ) were calculated based on OLS and WLS regression parameters. The relative error (ε_r_) obtained by OLS was 0.97%, 1.31%, and 0.52%, and by WLS was −0.25%, 1.11%, and 0.55%, for isopropanol, hydrochloric acidic medium, and phosphate buffer, respectively. The calculated relative standard deviation (RSD) ranged from 0.09% to 0.59%. The LOD and LOQ that were calculated by OLS were 0.59 mg/mL and 1.79 mg/mL, respectively. The LOD and LOQ that were obtained by WLS were 0.57 mg/mL and 1.73 mg/mL, respectively. Table 4 presents the obtained data. The validated UV method met all the requires parameters.

### 3.9. Drug Release Study

Phenylbutazone is a member of BCS class II that is characterized by high permeability and low solubility, limiting the absorption of PBZ in gastrointestinal tract. Importantly, the absorption of PBZ may be improved by increasing its dissolution rate [15]. 

In our study, we adsorbed PBZ on SBA-15-pr-NH_2._ The calculated amount of loaded PBZ obtained from the UV analysis was 5.04%.

**Figure 9:**
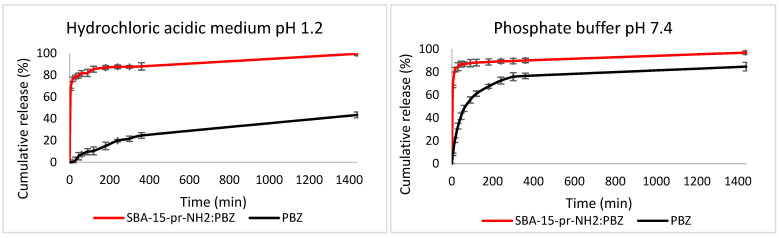
Dissolution profiles of SBA-15-pr-NH_2_:PBZ and PBZ.

Figure 9 demonstrates PBZ release profiles from mesoporous material at pH = 1.2 and pH = 7.4 compared to the dissolution of the crystalline PBZ. The release profiles of PBZ from SBA-15-pr-NH_2_:PBZ in both pH values are characterized by the initial burst release, followed by prolonged release rate. This type of release profile was also observed by Xu et al. [42]. In release studies of phenylbutazone from other carriers, such as PLGA nanocapsules, initial burst release was also observed [43]. In hydrochloric acidic medium 67% of PBZ was released in the first 5 min of the test, reaching almost 100% after 24 h. By contrast, the crystalline PBZ dissolved only in 43% within 24 h. In phosphate buffer at pH = 7.4, the differences are less pronounced but still significant. Similarly, in the first 5 min of the test, 67% of PBZ was released from the SBA-15-pr-NH_2_, whereas the phenylbutazone dissolved only in 8%. The initial burst release may result from the presence of PBZ adsorbed at outer part of the SBA-15-pr-NH2 channels, whereas the sustained release of PBZ is associated with PBZ loaded within silicas’ pores or electrostatically attracted to the aminopropyl groups present on modified material. This may be due to the fact that the diffusion of PBZ adsorbed at the outer part of silica is faster than from the inside of the materials’ pore [15,44,45]. Significant improvement in the dissolution rate of PBZ may be due to the property of SBA-15 to provide a more soluble amorphous form of adsorbed drugs [6]. We confirmed the amorphous state of PBZ in the XRD and DSC analyses. The calculated similarity coefficients f_2_ (Table 5) indicate that the release rate of PBZ from SBA-15-pr-NH_2_:PBZ was higher than its dissolution rate. It is also worth noting that the PBZ release from mesoporous carriers remains constant in both pH values, proved by the value of the similarity coefficient f_2_ above 50.

The obtained results demonstrate that the adsorption of PBZ on APTES-modified SBA-15 increases the dissolution rate of the drug, regardless the pH value.

### 3.10. Cytotoxicity Studies

**Figure 10:**
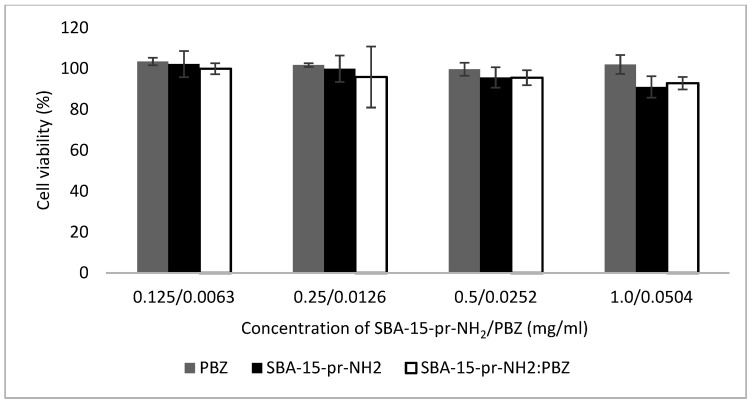
Caco-2 cell viability results after 2 h incubation with SBA-15-pr-NH_2_, SBA-15-pr-NH_2_:PBZ, and PBZ at 37 °C.

The biocompatibility of orally administered drugs, such as PBZ, and mesoporous silicas (SBA-15-pr-NH_2_, SBA-15-pr-NH_2_:PBZ) can be assessed using cell visibility tests. The test assumes that the amount of ATP (adenosine triphosphate) produced by metabolically active cells is proportional to the number of viable cells. In our study, we used Caco-2 cells (human colorectal adenocarcinoma cells), which are similar to the intestinal epithelial cells regarding their morphology and physiology [46]. Figure 10 shows the viability of Caco-2 cells after 2 h of exposure to PBZ (0.0063, 0.0126, 0.0252, 0.0504 mg/mL), SBA-15-pr-NH_2_ and SBA-15-pr-NH_2_:PBZ (0.125, 0.25, 0.50, 1.0 mg/mL). Our study showed no detrimental effect of analyzed substances on Caco-2 cells at the tested concentrations after 2 h of exposure. The cell viability did not differ significantly between samples, which allows us to assume that loading of PBZ to SBA-15-pr-NH_2_ did not influence the cytotoxicity of the material itself. Presented results are in agreement with literature data [47].

## 4. Conclusions

In this study, we analyzed the possibility of improving the dissolution rate of the phenylbutazone, a poorly soluble drug, by loading on APTES-modified mesoporous silica. We successfully synthesized the SBA-15 silica and modified its surface with 3-aminopropyl groups. We proved that proposed method of PBZ adsorption ensures the amorphous state of loaded drug, which was confirmed in XRD, DSC, SEM, and ^1^H-NMR analyses. Our results demonstrate increased dissolution rate of silica-loaded PBZ at various pH values compared to crystalline PBZ. Furthermore, contrary to the crystalline phenylbutazone, the release of amorphous PBZ from the mesoporous materials is not pH-dependent. Moreover, we revealed the negligible cytotoxicity of SBA-15-pr-NH_2_ before and after adsorption of PBZ. Concluding, the APTES-modified SBA-15 can serve as a non-toxic carrier for phenylbutazone improving its bioavailability.

## Figures and Tables

**Table 1:** Textural parameters of SBA-15, SBA-15-pr-NH_2_, and SBA-15-pr-NH_2_:PBZ.

Sample	Specific Surface Area (SBET) (m^2^/g)	Pore volume (Vp) (cm^3^/g)	Pore Diameter (nm)
SBA-15	789	1.021	6.0
SBA-15-pr-NH_2_	639	0.995	5.7
SBA-15-pr-NH_2_:PBZ	455	0.852	5.5

**Table 2:** The activation parameters of internal group reorientation of PBZ and SBA-15-pr-NH_2_:PBZ. The values of the uncertainty of the estimated parameters were lower than 10%.

Sample	1 Motion	2 Motion	3 Motion
PBZ	$\tau_{01}$ $\;=3.8\cdot 10^{-13}\left(s\right)$ $E_{a1}=16.0\;\left(\mathrm{kJ}/\mathrm{mol}\right)$ $∆M_{1}$ $\;=0.5\;\left(10^{-8}T^{2}\right)$	$\tau_{02}$ $\;=8.2\cdot 10^{-13}\left(s\right)$ $E_{a2}$ =9.8 (kJ/mol) $∆M_{2}$ $\;=1.3\;\left(10^{-8}T^{2}\right)$	$\tau_{03}$ $\;=3.9\cdot 10^{-13}\left(s\right)$ $E_{a3}$ =8.8 (kJ/mol) $∆M_{3}$ $\;=1.1\;\left(10^{-8}T^{2}\right)$
SBA-15-pr-NH_2_:PBZ	$\tau_{01}$ $\;=4.3\cdot 10^{-15}\left(s\right)$ $E_{a1}=24.8\;\left(\mathrm{kJ}/\mathrm{mol}\right)$ $∆M_{1}$ $\;=2.6\;\left(10^{-8}T^{2}\right)$	$\tau_{02}$ $\;=5.8\cdot 10^{-11}\left(s\right)$ $E_{a2}$ =3.7 (kJ/mol) $∆M_{2}$ $\;=5.4\;\left(10^{-8}T^{2}\right)$	$\tau_{03}$ $\;=7.8\cdot 10^{-11}\left(s\right)$ $E_{a3}$ =1.8 (kJ/mol) $∆M_{3}$ $\;=14.8\;\left(10^{-8}T^{2}\right)$

**Table 3:** Regression parameters of the analytical curve and sums of the relative errors (Σ%RE).

Regression	Isopropanol					HCl Medium (pH 1.2)				Phosphate Buffer (pH 7.4)			
	w_i_	a	b	r	∑ %ER	a	b	r	∑ %ER	a	b	r	∑ %ER
**Ordinary least squares**	1	51.37	0.0170	0.9999	3.81	48.94	0.0169	0.9991	6.76	38.06	0.0151	0.9995	4.23
**Weighted least squares**	$\frac{1}{y^{0.5}}$	51.29	0.0049	0.9999	1.38	49.17	0.0130	0.9991	5.49	37.97	0.0169	0.9995	0.59
	$\frac{1}{y}$	51.21	0.0058	0.9999	0.04	48.94	0.0170	0.9991	6.76	37.91	0.0178	0.9995	2.22
	$\frac{1}{y^{2}}$	51.07	0.0070	0.9999	0.17	48.92	0.0169	0.9908	5.60	37.90	0.0179	0.9995	1.66
	$\frac{1}{x^{0.5}}$	48.88	0.0179	0.9991	43.96	48.88	0.0179	0.9908	9.63	37.97	0.0169	0.9995	1.03
	$\frac{1}{x}$	51.21	0.0059	0.9999	0.19	48.90	0.0176	0.9908	8.52	37.92	0.0178	0.9995	2.80
	$\frac{1}{x^{2}}$	51.07	0.0070	0.9999	0.18	49.23	0.0144	0.9905	7.26	37.92	0.0177	0.9995	2.76

**Table 4:** Accuracy and precision of the UV method.

Concentration (mg/mL)	Accuracy (%) (Expressed as $\varepsilon_{r}=\frac{C_{theoret\;}-C_{exp}}{C_{theoret}}\times 100{\%}_{.}$) (*n* = 6) Acceptance Limit: ε_r_ < 5%		Precision (%) (Expressed as RSD, *n* = 6) Acceptance Limit: RSD < 5%	LOD (mg/mL)	LOQ (mg/mL)
	OLS $w_{i}\;=\;1$	WLS $w_{i}\;=\;\frac{1}{y}$			
**Isopropanol**	0.97	−0.25	0.09	3.20 × 10^−4^	9.70 × 10^−4^
**HCl medium (pH 1.2)**	1.31	1.11	0.59	1.13 × 10^−3^	3.41 × 10^−3^
**Phosphate buffer (pH 7.4)**	0.52	0.55	0.35	1.04 × 10^−3^	3.16 × 10^−3^

**Table 5:** Comparison of the similarity coefficients f2 between SBA-15-pr-NH_2_:PBZ and crystalline PBZ and between mesoporous silica in various pH.

	PBZ (pH 1.2)	SBA-15-pr-NH_2_:PBZ (pH 7.4)	PBZ (pH 7.4)
SBA-15-pr-NH_2_:PBZ (pH 1.2)	7.66	66.21	-
SBA-15-pr-NH_2_:PBZ (pH 7.4)	-	-	21.54

## Data Availability

The data presented in this study are available on request from the corresponding author.
